# Qualitative analysis of hotspots and intrusive memories after viewing an aversive film highlights their sensory and spatial features

**DOI:** 10.1038/s41598-022-10579-0

**Published:** 2022-04-29

**Authors:** Laura Singh, Brianna Garate, Johanna M. Hoppe, Emily A. Holmes

**Affiliations:** 1 Department of Psychology, Uppsala University, Box 1225, 751 42 Uppsala, Sweden; 2 Swedish Collegium for Advanced Study, Uppsala, Sweden; 3 Department of Medical Sciences, Uppsala University, Uppsala, Sweden; 4 Division of Psychology, Department of Clinical Neuroscience, Karolinska Institutet, Stockholm, Sweden

**Keywords:** Psychology, Human behaviour

## Abstract

Intrusive memories of trauma are recurrent distressing sensory-perceptual impressions of the traumatic event that enter consciousness spontaneously and unwanted. They often contain the worst moment/s (‘hotspots’) of the trauma memory and have primarily been studied in clinical populations after real trauma. Intrusive memories can also be studied using analogue trauma as an ‘experimental psychology model’. Little is known about the features of analogue trauma hotspots. Here we report an ancillary analysis of data from a randomized controlled trial. Seventy non-clinical participants viewed a trauma film containing COVID-19 related footage. Features of hotspots/intrusive memories of the film were explored using linguistic analysis and qualitative content coding. Participants reported on average five hotspots (*M* = 9.5 words/hotspot). Akin to hotspots soon after real trauma, analogue hotspots/intrusions primarily contained words related to space. Most contained sensory features, yet few cognitions and emotions. Results indicate that features of analogue trauma hotspots mirror those of hotspots soon after real trauma, speaking to the clinical validity of this ‘experimental psychology model’.

**ClinicalTrials.gov ID**: NCT04608097, registered on 29/10/2020.

## Introduction

Intrusive memories are recurrent, involuntary sensory-perceptual impressions (primarily visual) of a traumatic event that enter consciousness spontaneously and are a core clinical feature of posttraumatic stress disorder (PTSD; Refs.^1,2^). They can be distressing in their own right^3^ and impair functioning (e.g., disrupt concentration^4^). Intrusive memories typically include ‘worst moment’ scenes of the trauma (i.e., ‘hotspots’). The content of hotspots and intrusive memories reported by individuals with PTSD appears to be closely matched (e.g. 83% and 78% overlap^5,6^, respectively), though matching in those studies was done retrospectively.

Intrusive memories can be studied outside of clinical settings using an experimental analogue of trauma (e.g. the trauma film paradigm) in a controlled laboratory environment^7,8^. Such an ‘experimental psychology model’^9,10^ uses experimental approaches to model abnormal processes (e.g. intrusive memories). The trauma film paradigm has been used to develop novel interventions to reduce intrusive memories in non-clinical participants, before translation to individuals who experienced ‘real’ trauma^7,11^. A critique about analogue trauma studies important to translation concerns their ecological validity.

Even though the trauma film paradigm has been used to investigate intrusive memories in a large number of studies, only one study has assessed *hotspots* of analogue trauma in non-clinical participants to date. Using a virtual reality adaptation of the trauma film paradigm (i.e., a simulated terrorist attack in the Middle East), Nielsen et al.^12^ investigated characteristics (e.g. self-rated distress) of analogue trauma hotspots before and after participants recorded intrusive and voluntary memories in a 1-week daily diary. Most memories (intrusive and voluntary) matched previously identified hotspot moments, and hotspots that intruded were more distressing than those that did not intrude^12^.

Nielsen et al.^12^ analysis focused on the match between hotspot content immediately after analogue trauma (rather than retrospectively as in most clinical studies) and memory content during the subsequent week. Being the first to assess hotspots of analogue trauma, this study takes a crucial step towards exploring the clinical validity of analogue trauma research and leads to important further questions regarding differences and similarities between hotspots of analogue trauma and real trauma. One way to further enhance our understanding of analogue trauma hotspots is to explore their content qualitatively (e.g., using linguistic analysis and content coding). Qualitative content coding has often been used to explore hotspot characteristics in the clinical literature ^13–15^ but, to the best of our knowledge, has not yet been applied to analogue trauma hotspots.

In the current study we aim to address this important gap by mirroring the qualitative content analysis approach used in the clinical literature (real trauma) with hotspots of analogue trauma, here a film. Specifically, we use linguistic analysis and an adapted qualitative coding framework from our recent ‘real’ trauma hotspots study^16^. In this study, patients who experienced a traumatic event reported hotspots soon after in the Emergency Department. Linguistic analysis revealed that the majority of words in hotspots were related to time, space, motion, and sensory processing—a striking result as the focus on space/motion was not expected. Qualitative content analysis confirmed that 97% of hotspots contained sensory features (e.g., ‘*seeing* smoke from the car and airbag’) and 59% contained motion features (e.g., ‘I’m *flying* through the air toward the tree’), whereas only few cognitions and no emotions were identified. These findings suggest that hotspots/intrusions are mental imagery bound representations, at least in the early hours after trauma. Note that in contrast to most clinical work (e.g., Grey & Holmes and Holmes et al.^5,6^), both Hoppe et al.^16^ and the current analogue trauma study explore hotspots in the first hours after trauma.

Taking an established method (i.e., qualitative content coding) used to explore features of real trauma hotspots back to the laboratory can address limitations of previous clinical work: (1) in analogue trauma studies all participants are exposed to the same trauma film, allowing to explore both the participants ‘subjective’ description of a specific hotspot moment (i.e. the way they perceived and describe an image/clip from the film) as well as the ‘objective’ content of a specific hotspot moment (i.e. the actual content of the image/clip from the film); (2) analogue trauma models facilitate the assessments of hotspots immediately after trauma and before intrusive memories have formed, in contrast to clinical studies often relying on retrospective reports; (3) a larger number of participants can be easily assessed when investigating non-clinical samples rather than patient populations.

### Study aims

Aim 1: The first aim was to conceptually replicate/extend findings from our study exploring features of hotspots soon after real trauma, in an experimental setting using analogue trauma. Mirroring previous work, we explored frequency, sensory-perceptual features and other content of hotspots collected immediately after analogue trauma using linguistic analysis and qualitative coding (coding framework adapted from Hoppe et al. and Holmes et al.^6,16^. Aim 2: The second aim was to extend previous investigations by exploring similarities and differences between features of hotspots reported immediately after analogue trauma and intrusive memories formed later on, using the same linguistic analysis and qualitative coding as for hotspot data. Aim 3: We also aimed to explore the overlap of specific clips within the trauma film described in hotspots and intrusive memories, that is, (1) to what extent the same clips described as hotspots are later reported as intrusive memories and (2) the proportion of intrusive memories that contain clips also described as hotspots.

## Materials and methods

In the current analogue trauma study, hotspot data were collected in the context of a randomized two-group experimental trial investigating the feasibility of delivering the trauma film paradigm, a brief cognitive intervention/control task, and a daily intrusion diary remotely/digitally during the COVID-19 pandemic (ClinicalTrials.gov ID: NCT04608097, registered on 29/10/2020)^17^. All parts of the experimental protocol were based on previous studies conducted in a laboratory in the Psychology Department at Uppsala university and have been translated for remote delivery with researcher guidance in a ‘digital lab’ to adhere to guidelines on social distancing during the COVID-19 pandemic (e.g., participants participated online from their homes; questionnaires and diary were assessed electronically; instructions were digitalized using videos of a researcher or animated videos, and participants’ understanding of instructions was confirmed during brief phone/video calls with the researcher in the end of the online session).

### Participants

A total of 74 non-clinical participants were recruited via social media advertisements (e.g. groups for Swedish university students) between October and March 2021 (see Supplementary Fig. 1 for a participant flow diagram). Inclusion criteria were: aged 18–65; Fluent in spoken and written Swedish; Willing to watch a video containing emotional, distressing footage; Having access to an internet enabled smartphone/computer. Exclusion criteria were: Having participated in a study in which similar stimuli were used; currently receiving treatment for a mental health problem (e.g., depression, anxiety, ADHD, addiction), including psychological therapy, counselling or medication; Neurological illness (e.g., epilepsy); Planning to undertake a stress-inducing examination (e.g., university examination or driving test) during the week of study participation.

The sample size was determined for the wider intervention study using an apriori power calculation estimating that at least 30 participants per group were required to detect a mean effect size of *d* = 0.962 (mean between-group effect size of previous similar studies). Four participants were excluded from analyses because correct protocol completion could not be confirmed in the digital setting, leaving a final sample of 70 participants (female n = 47, male n = 23, transman/transwoman/genderqueer/non-binary/other identity: n = 0, age: *M =* 23.89 years, *SD =* 4.86 years). Recruitment stopped once the required minimal sample size per group had been reached. All participants provided hotspot and intrusive memory data digitally. The primary outcome data of the current study is the participant’s written descriptions of hotspots and intrusive memories.

### Design

This study used a mixed methods design, combining linguistic analysis, qualitative content coding and quantitative analysis.

### Materials

#### Trauma film

The 11:22-min film comprised 12 different short clips depicting serious illness, violence, and death in relation to the COVID-19 pandemic. All clips were available in the public domain (i.e. news footage and documentary footage) and were compiled by the study team. In the beginning of the film, three written statements about the COVID-19 pandemic (e.g., ‘COVID-19 takes lives in all ages groups. Young and healthy people have died from the disease.’) were presented for 4 s each as white text on a black screen. Each clip was introduced with a written statement presented for 4 s (e.g. ‘22-year old Amin’s unexpected death from COVID-19 is mourned by family and friends’). The film was played via Vimeo (InterActiveCorp, New York, USA) on the participant’s own computer. Participants were instructed to view the film alone in a dark room in full screen mode, sit close to the screen to make sure it filled their entire field of view, listen to the audio with headphones and increase the volume to a bit louder than usual. The majority of participants (i.e., 94–99%) indicated that they adhered to these instructions.

#### Hotspot list

Hotspot data was collected in the digital data collection platform Qualtrics (Provo, USA). Participants were instructed (by video of a researcher and written instructions) to list their worst moment images of the film (e.g. ‘The dead man on the street’). Specifically, they were asked to think back to the film and briefly describe each worst moment image that came to mind (i.e., images that they saw in the film or related images they imagined while watching the film). They did not need to think about them in detail, but could describe in a few words which images came up from these moments. The digital platform provided space for six different hotspot images and participants were instructed to list all remaining images in the final space if they had more than six hotspots.

#### Intrusive memory diary

Intrusive memory data was collected in a daily diary for seven days, starting 24 h after film viewing. Both verbal (video of a researcher) and written instructions were given on how to complete the diary. Participant’s understanding of instructions about the definition of intrusive memories and how to complete the diary were checked by a researcher in a follow-up phone call. The diary was based on the pen-and-paper diary used in previous trauma film studies from our group^18–20^ but collected digitally (i.e., adapted for remote data collection during the ongoing COVID-19 pandemic). Instead of ticking a box for each intrusive memory on a pen-and-paper diary, participants received a link to a brief questionnaire sent automatically via email by Qualtrics (Provo, USA) each day. Participants recorded how many intrusive memories related to the film they experienced since film viewing (Day 1)/the last recording (Day 2–7) (possible answers were 0–15). Then participants were asked to briefly describe the content of each intrusive memory they had recorded. Separate content descriptions for each memory were required even if the same intrusion occurred several times. Intrusive memories of the film were defined as following: ‘Intrusive memories are: images or other sensory impressions from the COVID-19 film (or related images you imagined while viewing the film), that suddenly pop into mind involuntarily; They can be short and fleeting; They are NOT the same as deliberately choosing to think about specific images from the film. If you think about the film in general and a specific image pops into your mind without you wanting it, it is an intrusive memory; Intrusive memories are not the same as thinking about the film in words. Please record every intrusive memory you have had—even if it is the same one popping up several times. If you did not have any, please choose 0.’ If a participant had not completed the diary in the morning after receiving the link, they were reminded by a researcher.

### Procedure

#### Study procedures

After providing their written and informed consent digitally, participants completed baseline questionnaires (e.g. demographics) and practiced a task related to the intervention study from which the data of the current study were derived. Then participants viewed the trauma film. They were instructed to immerse themselves in the film, imagine they were bystanders witnessing the scene or that the events would happen to themselves or their family/friends. Following film viewing they provided hotspot data and completed the intervention or control task (not relevant for the current study). Last, participants received instructions about the definition of intrusive memories and how to complete the diary over the following 7 days. Detailed descriptions of all measures used in the intervention study from which the current data were derived are preregistered in a public registry (ClinicalTrials.gov ID: NCT04608097, registered on 29/10/2020).

#### Training of student researchers

Prior to data collection, researchers who interacted with participants received formalised training from a researcher experienced in delivering the protocol in the laboratory. The training included theoretical background about the procedures, practical training (e.g. role plays) on crucial parts of protocol delivery (e.g., how to provide a definition of hotspots and intrusive memories and check participants understanding) and continued supervision by LS and EAH throughout data collection.

### Data analysis

Two independent researchers (LS and BG) checked whether descriptions of hotspots and intrusive memories matched with the film content (i.e., whether the described content related to one of the film clips). Only hotspots and intrusive memories that could be matched to the film were analysed (though note that descriptions did not need to match the film content exactly, i.e., if a participant had added/changed details of the film in their hotspots/intrusive memory descriptions but it was possible to match them to the film, they were included; e.g. ‘my mother lying in a respirator’).

All quantitative analyses were performed using IBM SPSS Statistics for Windows, version 25 (IBM Corp., Armonk, NY).

#### Hotspot/intrusive memory frequency and linguistic features

Hotspot frequency (i.e. the number of hotspots of the film) was calculated by summing the number of hotspots reported per participant as listed in the hotspot list. If participants reported more than one hotspot in the last row of the list, these were counted as separate hotspots.

*Intrusive memory frequency* (i.e. the number of intrusive memories of the film) was calculated by summing the number of intrusive memories reported per participant over the 7-day digital diary. If participants listed intrusive memories of different moments of the film in one entry, they were separated. Because the wider study the data for the current study was drawn from included an intervention that may have affected intrusive memories, only participants from the control group (n = 37) were included in analyses of intrusive memory data (i.e., frequency and features of intrusive memories; and the overlap of scenes described in hotspots and intrusive memories).

The text analysis software ‘Linguistic Inquiry and Word Count’ (LIWC, 2015 for Windows, Pennebaker et al.^21^) was used to categorise the features of hotspots and intrusive memories based on the words used by participants to describe their hotspots and intrusive memories into predefined word categories (selected categories for the current study were ‘affective processes’, ‘cognitive processes’, ‘perceptual processes’, ‘biological processes’, ‘time orientation’ [i.e. past, present future] and ‘relativity’ [i.e. space, time and motion words]). See Hoppe et al.^16^ for a detailed description of LIWC analysis.

Hotspots and intrusive memory descriptions provided by the participants in Swedish were translated to English by a native Swedish and English speaker (BG), and subsequently used as input data for LIWC analyses. The mean number of words per hotspot and intrusive memory was extracted. The error rate (calculated through manual double-checking of 10% of coded data) of misclassified words was 9% for hotspot data (i.e., the word ‘lying’ was miscoded as related to *negative affect* and *anger*, when it in fact referred to lying down rather than dishonesty and the word ‘admit’ was miscoded as related to *insight*, when it in fact referred to hospital admission rather than conceding something as true or valid) and 3% for intrusive memory data (i.e., the word ‘over’ was miscoded as related to *space*, when it in fact referred to via [over/via the phone] rather than above).

#### Sensory-perceptual features and other content

The analysis of sensory-perceptual features was based on the hotspots coding frame used in Hoppe et al.^16^. The coding framework was designed to capture sensory-perceptual features and other imagery-related content in hotspots after real trauma and included five sensory-perceptual features (e.g., visual, proprioceptive-kinesthetic, auditory, tactile), and eight other content categories (e.g., narrative cohesiveness, content conveys threat, motion features; see Hoppe et al.^16^ for a detailed description of categories). Here, we adapted the coding framework to fit hotspots and intrusive memories after analogue trauma (i.e. ‘witnessing’ potentially traumatic events shown in a film) as opposed to self-experienced, real trauma. The adaptation entailed removing categories that were not possible to code due to the film format, for instance, the sensory-perceptual category ‘proprioceptive-kinesthetic’ (i.e., movement or change in the position of the body) and four content categories (‘narrative cohesiveness’, ‘first sign of threat’ ‘attempted action’, ‘outside body perspective’).

Furthermore, the category ‘motion features’ was adapted by defining motion or action as the presence of verbs associated with motion, e.g., lifting, screaming (in Hoppe et al.^16^, motion was simply defined as ‘hotspot including any motion’). Verbs used to describe a static scene, e.g., laying, sitting, were not coded as motion. Based on bottom-up reading of hotspots and intrusive memories, we added the new category ‘imagined content’ with the subcategories ‘self-relevant’ and ‘any’. These codes referred to imagined scenes where participants describe events from the trauma film in relation to the self being present/affected (e.g. the event happening to them or their family/friends) and to imagined scenes that included added, changed, or distorted content from the film. In addition, the category ‘crying—screaming’ was added to further examine auditory features in hotspots and intrusive memories. Lastly, examples in the coding frame were adapted to the trauma film (e.g., examples for the ‘body/biology’ category included ‘death’, ‘trouble breathing’).

#### Emotional and cognitive themes

The analysis of emotional and cognitive themes was based on the hotspots coding frame developed by Holmes et al.^6^ (also used by Hoppe et al.^16^). Coding of emotional themes included the following categories: anger, fear, disgust, sadness, happiness, shame, guilt, surprise, helplessness, horror and the state of dissociation (although not strictly an emotion). For example, if a participant reported feeling trapped, this was coded under ‘helplessness’; if they used the word ‘terrified’, it was coded under ‘fear’^6^. Based on bottom-up reading, subcategories were added to code whether emotions in hotspots/intrusive memories referred to emotions experienced by the participant (‘emotion—experienced’) or wether emotions were displayed by individuals in the film (‘emotion—witnessed’). Coding of cognitions included seven overarching cognitive themes as well as 21 coding categories: (1) uncertain threat (unease, confusion, realisation of a nonspecific threat, ongoing threat), (2) general threat of injury and death (self-dying, self will die, self injured, self will be injured, death or injury of others), (3) control and reasoning (interpersonal reasoning, planning, revenge/injustice), (4) consequences (consequences, relief, realisation after), (5) abandonment (let down by others, outrage), (6) esteem (self-blame/criticism), and (7) cognitive avoidance (disbelief, dissociation).

#### Coding

Coding was conducted at the level of each hotspot and intrusive memory. Given that descriptions were brief and could be best coded simply based on the presence or absence of each content category, coding was conducted in Microsoft Excel for Windows (2013) independently by two researchers (BG & JMH). Cohen’s kappa indicated substantial to perfect agreement (*κ* = 0.67–1.0). Any disagreements were discussed, and decisions about final category allocation were made jointly. The complete hotspot coding framework is presented in Supplementary Table S1.

#### Overlap of scenes described in hotspots and intrusive memories

Each hotspot and intrusive memory description was categorized into one of the 12 discrete clips within the film (e.g., clip 1: ‘Memorial of 22-year old Amin’, coding done by BG). Because some descriptions could not be matched to a specific clip, 4 more broader categories were created to cover descriptions that matched similar clips (e.g. clip 6 or 9: ‘Combination of clips with COVID-19 footage from Italian hospitals’). Categorization of descriptions to the different film clips was double checked for 10% of hotspots and intrusive memories by an independent researcher (LS). For hotspot data, a discrepancy rate of 11% (2 out of 18) was found. For intrusive memories, a discrepancy rate of 7% (2 out of 30) was found.

To explore the overlap between clips described in hotspots and intrusive memory data, we computed the following variables for each participant included in this analysis (n = 37): (1) the number of hotspots that were also reported as intrusive memories later on and (2) the number of intrusive memories that contained scenes also previously described as hotspots. Then, we calculated the following proportions: (1) the total number of hotspots that were also reported as intrusive memories across participants divided by the total number of hotspots reported across participants and (2) the total number of intrusive memories that were also previously described as hotspots across participants divided by the total number of intrusive memories reported across participants.

### Ethical considerations

Ethical approval for the current study was embedded in the approval obtained for the overarching study in which the data analysed here was collected. The ethics committee of the Swedish Ethical Review Authority approved the study (approval number: 2020-03991). All methods were carried out in accordance with the ethical guidelines of the Declaration of Helsinki. All participants provided their written and informed consent prior to starting the study. The study was pre-registered on clinicaltrials.gov prior to study start (ID: NCT04608097, registered on 29/10/2020). The full trial protocol can be found in the Supplementary Information. Data were handled confidentially and according to the General Data Protection Regulation (GDPR).

## Results

A total of 339 hotspots were reported, of which 2 (0.6%) could not be matched to the film. Thus, analysis of hotspot data included 337 discrete hotspots recorded from all 70 participants (Aim 1). A total of 308 intrusive memories were reported in the control group of which 4 (1.3%) could not be matched to the film. Because of a technical error, we did not obtain content descriptions for 8 (2.6%) intrusive memories. Thus, analysis of intrusive memory data included 296 discrete intrusions recorded from the 37 participants in the control group [Aim 2; note that 8 (21.6%) participants did not experience any intrusive memories]. As the study focused on the features of hotspots and intrusive memories per se, parts of descriptions that merely described triggers (e.g., ‘I thought about the film in general terms and …’; ‘Thought about ghosts and…’) or commented a film clip (e.g., ‘That was difficult to watch’; ‘That was probably the most difficult’) were removed (hotspots: n = 2; intrusive memories: n = 6; see Supplementary Table S2). De-identified summary data and codebook are available on the Open Science Framework: https://osf.io/4zqkm/.

### Hotspot frequency and linguistic features

Participants reported a mean of 4.8 hotspots (*Mdn =* 5.0, *SD =* 1.4, range from 3 to 11). Word count extracted with LIWC software showed that hotspots included a mean of 9.5 words. The percentages of words in hotspots associated with specific word categories of interest are presented in Table 1. The most common word category was ‘relativity’ words—i.e., words related to space, time and motion, followed by ‘affective processes’ words—i.e., those related to positive/negative emotion, anxiety, anger and sadness. Full LIWC results can be found in OSF (https://osf.io/4zqkm/) and more examples of words coded into each word category are presented in Supplementary Tables 3 and 4.

**Table 1:** Percentages of words within hotspots/intrusive memories matching LIWC word categories of interest in linguistic analysis.

LIWC^a^ word category	Hotspots	Intrusive memories	Example words
	%	%	
**Relativity**	17.23	17.51	
Motion	0.87	0.46	Removed
Space	13.61	15.36	Street
Time	2.55	1.74	Young
**Perception**	3.84	4.17	
See	0.69	1.45	Image
Hear	2.94	2.67	Screaming
Feel	0.18	0.06	Pain, feeling
**Biological processes**	4.88	4.64	
Body	1.29	1.28	Bodies, head
Health	3.42	3.01	Hospital, sick
Sexual	0.03	0.00	Naked
Ingestion	0.18	0.35	Dining
**Time orientation**			
Time—past	3.54	1.80	Dies
Time—present	5.24	3.01	Has
Time—future	0.45	0.23	Will, going
**Affective processes**	5.93	3.01	
Positive emotion	0.81	0.35	Party
Negative emotion	5.09	2.67	Ignored
Anxiety	0.84	0.35	Panic
Anger	1.05	0.41	Violent
Sadness	1.05	0.35	Cry
**Cognitive processes**	3.27	1.97	
Insight	0.48	0.35	Memory
Causation	0.45	0.29	Because
Discrepancy	0.33	0.00	Wished
Tentative	0.63	0.52	Seems
Certainty	0.45	0.00	Clear
Differentiation	1.23	0.93	Against

Analysis of hotspots data included 337 discrete hotspots recorded from 70 participants. Analysis of intrusive memories included 296 discrete intrusive memories from 37 participants (only intrusive memories reported by participants in the control group were analysed because the intervention group explored in the wider project the current data was collected in might have influenced intrusion data). Analyses were conducted using the ^a^Linguistic inquiry and word count (LIWC 2015) software. % = percentage of words within hotspots/intrusive memories matching LIWC word categories and subcategories. Last column provides examples of words within hotspots/intrusive memories categorised into LIWC word categories. Misclassified words are not presented (e.g., lying, over). For comparison, categories are presented in the same order as Hoppe et al.^16^.

### Sensory-perceptual features and other content of hotspots

Nearly all hotspots contained sensory features (99.7%), the most common sensory modality reported being visual hotspots (e.g., ‘The *images* of the young girl.’). However, auditory features were also described frequently (28.5%). Most auditory hotspots (74.0%) referred to people crying, screaming or yelling (e.g., ‘The nurse who is *screaming* and *crying*.’). Other features of hotspots included content that conveys threat (e.g., ‘Police who are *violent* toward people.’), motion features (e.g., ‘When they *dragged* out people from their homes.’) and content related to the body or biological processes (e.g., ‘*Dead bodies* in the corridor of the hospital.’). Some hotspots contained imagined content, such as added, changed or distorted content from the film (e.g., ‘A *bloody* circular saw.’ [the film depicted a drawing of a circular saw without blood]), or events from the film described in relation to the self being present or affected (e.g., ‘*My father or mother* lying dead with COVID-19.’). Percentages of sensory-perceptual features and other content of hotspots are presented in Table 2.

**Table 2:** Frequency of sensory-perceptual features, content and emotional themes in hotspots and intrusive memories.

	Hotspots		Intrusive memories	
	n	%	n	%
**Sensory features**				
Visual	333	98.8	289	97.6
Auditory	96	28.5	53	17.9
Crying—screaming	71	21.1	31	10.5
Tactile	7	2.1	0	0
No sensory features	1	0.3	2	0.7
**Content features**				
Content conveys threat	289	85.5	181	61.1
Any motion features	186	55.2	91	30.7
Body/biology	177	52.5	103	34.8
Imagined content	36	10.7	40	13.5
Self-relevant	15	4.5	14	4.7
Any	21	6.2	26	8.8
**Cognitive and emotional themes**				
Cognition—any	6	1.8	5	1.7
Uncertain threat	3	0.9	2	0.7
General threat of injury/death	0	0	1	0.3
Abandonment	0	0	1	0.3
Esteem	1	0.3	0	0
Emotion—any	39	11.6	8	2.7
Emotion—witnessed	37	11.0	7	2.4
Emotion—experienced	2	0.6	1	0.3
Fear	12	3.6	3	1.0
Helplessness	15	4.5	3	1.0
Sadness	5	1.5	1	0.3
Horror	3	0.9	0	0
Guilt	1	0.3	0	0
Surprise	1	0.3	0	0
Anger	1	0.3	2	0.7

Analysis of hotspots data included 337 discrete hotspots recorded from 70 participants. Analysis of intrusive memories included 296 discrete intrusive memories from 37 participants (only intrusive memories reported by participants in the control group were analysed because the intervention group explored in the wider project the current data was collected in might have influenced intrusion data). Only emotional and cognitive themes identified in the dataset are presented here. For comparison, categories are presented in the same order as Hoppe et al.^16^.

n = number of hotspots/intrusive memories with feature or theme.

% = percentage of the total number of hotspots/intrusive memories with feature or theme.

### Emotional and cognitive themes in hotspots

Using the coding frame developed by Holmes et al.^6^, some hotspots contained reference to emotion (11.6%), mostly describing emotions observed in the film (94.8% of reported emotions) rather than emotions experienced by the participant (5.2% of reported emotions, e.g., ‘I am standing in a *panic* and speaking on the phone.’). The most common emotion categories identified were helplessness (e.g., ‘*Hopeless* woman shouts for help.’) and fear (e.g., ‘The doctor that is in *panic*.’). Only six cognitions were identified, which included the themes uncertain threat (e.g., ‘Young people who die *that could just as well have been me*.’) and esteem (i.e., ‘The house parties. Guilt that *I have not taken everything seriously during the entire pandemic*.’).

### Intrusive memory frequency and linguistic features

Participants (control group only) reported a mean of 8.2 intrusive memories (*Mdn =* 4.0, *SD =* 9.9, range from 0 to 46) during the week. Word count extracted with LIWC software showed that intrusive memories included a mean of 5.8 words. The percentages of words in intrusive memories associated with specific word categories of interest are presented in Table 1. The most common word category was ‘relativity’ words—i.e., words related to space, time and motion, followed by ‘biological processes’ words—i.e., those related to body, health, sexual processes and ingestion.

### Sensory-perceptual features and other content of intrusive memories

Similar to hotspots, nearly all intrusive memories contained sensory features (99.3%), mostly visual (e.g., ‘The *image* of the man who spoke indignantly on the phone.’) and auditory features (e.g., ‘The staff who *screams* on the phone.’), though auditory features were reported to a lesser extent in intrusive memories than hotspots (17.9% vs. 28.5%). The most common other features of intrusive memories were (also in line with hotspots data) content that conveys threat (e.g., ‘Healthcare staff is in a *panic*.’), motion features (e.g., ‘People are *dragged out*.’) and content related to the body or biological processes (e.g., ‘*Dead* people in hospital corridor.’). Intrusive memories contained slightly more imagined content than hotspots (13.5% vs. 10.7%, e.g., ‘The dead *people* on the street.’ [only one dead body on the street was depicted in the film] or ‘*Mother* in respirator.’). See Table 2 for details.

### Emotional and cognitive themes in intrusive memories

Only 8 intrusive memories contained reference to emotion (2.7% compared to 11.6% in hotspot data), again mostly referring to witnessed emotions, i.e., emotionsexpressed by people in the film (88.9% of reported emotions) rather than self-experienced (11.1% of reported emotions). The most common emotion categories identified were fear (e.g., ‘The woman who is screaming in China alone on a balcony.’) and helplessness (e.g., ‘The doctor who quit in a panic.’). Akin to hotspot data, very few cognitions were identified. They included the themes uncertain threat (e.g., ‘Staff member at a hospital in Italy, overwhelming COVID-19 and they do not have any solution’) and general threat of injury/death (i.e., ‘That they are dragged away/carried away from their home by multiple people, one of them struggles with their whole body not to be removed—I imagine that what is waiting them is perhaps death or some type of quarantine where they will be left to die.’) and abandonment (i.e., ‘That healthcare staff explained the situation at the hospital on the phone and did not seem to get any respect for how serious the current situation was.’).

### Overlap of clips described in hotspots and intrusive memories

(1) 35.8% of hotspots referred to clips of the film that were also reported in intrusive memories in the following week. (2) 61.8% of intrusive memories (proportion between the number of times a clip, that was previously described as a hotspot, intruded and the total number of intrusive memories) had previously been described as a hotspot. The clips most commonly described as hotspots were of an overcrowded hospital in China displaying dead and seriously ill patients lying on the hospital floor and of Chinese hospital staff crying and screaming in distress in the break room and yelling at their supervisors that they want to quit their job. The clips most commonly described as intrusive memories were of a woman who is standing on the balcony and desperately screaming for help because her mother is dying and of an overcrowded hospital in Italy showing many seriously ill patients with ‘plastic bubbles’ (ventilators) over their head and hospital staff being overwhelmed by the amount of work.

## Discussion

This study is the first to investigate features of analogue trauma hotspots and intrusive memories qualitatively as in previous clinical work on real trauma. Linguistic analysis revealed that relativity words (space: ‘dead patients on the hospital *floor’*), dominated in hotspot/intrusive memory descriptions, which is in line with findings from a recent study on hotspots soon after real trauma^16^. Also in line with real trauma hotspots, qualitative content coding of analogue trauma hotspot/intrusive memory descriptions demonstrated that sensory (visual/auditory: ‘The *images* of the young girl.’, ‘The nurse who is *screaming* and *crying*.’) and motion features (‘When they *dragged* out people from their homes’) were most common in addition to content that conveyed threat and body/biology features.

Hoppe et al.^16^ found that recently encoded hotspots of real trauma were predominantly expressed as sensory-based experiences, specifically including visuospatial and motion features, whereas few hotspots referenced to cognitions and none to emotion. Even though the trauma content in Hoppe et al.^16^ (e.g. fall accidents) differed from the current analogue trauma study (COVID-19 related film footage), similar proportions of word categories emerged from linguistic analysis of hotspot descriptions: the most common word category identified was ‘space’ (e.g., falling, floor; 14% and 18% of words in hotspots in the current study and Hoppe et al.^16^, respectively). However, real trauma hotspots contained more perception words (e.g., look, hear; 11%) than analogue trauma hotspots (4%), whereas more affective words (e.g. violent, panic) were identified in analogue trauma hotspots (6% vs. 2% in Hoppe et al.^16^). This discrepancy might arise from differences between *witnessing* analogue trauma, which comprises highly emotional footage, and *experiencing* real trauma.

Qualitative content coding of analogue trauma hotspots also largely mirrored findings from real trauma: sensory features were most common, with visual features being identified in 99% of analogue trauma hotspots compared to 76% of real trauma hotspots. Interestingly, analogue trauma hotspots included more auditory features (29% compared to 10% in Hoppe et al.^16^), mostly referring to crying, screaming or yelling—sounds related to survival from an evolutionary perspective. The majority of hotspots in both studies contained content conveying threat (86% and 71% here and in Hoppe et al.^16^) and slightly more than half of hotspots contained motion features (55% and 59% here and in Hoppe et al.^16^, respectively). These similarities between hotspots of analogue and real trauma speak to the clinical validitiy of the trauma film paradigm as an experimental psychology model^9^.

A new content coding category in the current analogue trauma study (based on bottom-up reading of hotspot/intrusive memory descriptions) included hotspots containing imagined content (‘*My father or mother* lying dead with covid-19’). Around 11% of hotspots and 14% of intrusive memories in the current study contained imagined content. Note that participants were instructed to immerse themselves during film viewing and imagine they were bystanders directly witnessing the events in the film or that the events were happing to themselves or their friends/family. There is also some evidence of imagined content in intrusive memories in the clinical literature: Holmes et al. (2007) reported that after having viewed the attacks of September 11, 2001 on television, London school children experienced intrusive memories containing imaginary scenes (‘I saw my uncle trying to get out’)^22^. Such hints on imagined content in hotspots and intrusive memories of analogue and real trauma can be important for the development of future treatments following trauma exposure.

We also collected descriptions of intrusive memories reported in the week after analogue trauma and could therefore apply the same analyses as used for hotspots to intrusive memory data (Aim 2). Interestingly, in comparison to hotspots, intrusive memories included even more words related to space and perception, whereas fewer words related to biological, affective, and cognitive processes were identified by linguistic analysis. Qualitative content coding revealed that intrusive memories contained about the same proportion of visual features (99% in hotspots vs. 98% in intrusive memories) and more imagined content (11% in hotspots vs. 14% in intrusive memories), whereas a smaller proportion of other content categories was identified (i.e., fewer auditory, motion, body/biological features, less content that conveys threat and fewer cognitions/emotions in intrusive memories than hotspots). One explanation for this difference, is that intrusive memory descriptions were, in general, briefer than hotspot descriptions (*M* = 5.8 words per intrusive memory vs. 9.5 words per hotspot) and often focussed precisely on visual features of the image content.

We also compared the overlap of moments described in hotspots reported immediately after film viewing and intrusive memories reported later on (Aim 3). About 36% of hotspot clips became intrusive—a higher proportion than the 12% reported in Nielsen et al.^12^ though note that their participants reported fewer intrusive memories in general. About 62% of intrusive memories in the current study had been previously reported as hotspot moments (see also Supplementary Results), which is comparable with Nielsen et al.^12^ findings (66% of intrusive memories overlapped with hotspot content), though surprising in relation to studies with PTSD patients (i.e., 83% and 78% overlap^5,6^). One explanation for this discrepancy is methodological differences—while hotspots were assessed retrospectively in real trauma studies, both analogue trauma studies assessed hotspots immediately after trauma and before intrusive memories had formed, thus possibly leading to more precise proportions.

Limitations of the current study include that (1) instructions might have influenced some of the results (e.g. participants were instructed to describe *images* of the film in their hotspots/intrusive memories, which might have steered them towards describing imagery-related scenes). However, this cannot explain findings related to relativity or motion features; (2) the analogue trauma content (COVID-19 related footage in e.g. hospitals) differed from the types of traumatic events experienced by participants in Hoppe et al.^16^ (e.g., motor vehicle accidents), which limits the extent to which some qualitative features can be compared—future studies should compare the results from the current COVID-19 analogue trauma study with studies exploring hotspots of real trauma related to the COVID-19 pandemic, e.g. in healthcare staff exposed to trauma at work during the pandemic^23^.

Strengths of the current analogue trauma include (1) a larger sample compared to previous work on analogue^12^ and real trauma^5,6,16^ and (2) that all participants were exposed to the same (analogue) trauma, allowing us to explore participants ‘subjective’ description of a specific hotspot moment (i.e. the way they perceived/described an image/clip from the film) and the ‘objective’ content of a specific hotspot moment (i.e. the actual content of the image/clip from the film), which is seldomly possible for real traumatic events. This led, for instance, to novel findings regarding imaginary content of hotspots and intrusive memories.

In conclusion, the current study shows that hotspots after analogue trauma display similar linguistic features and content as hotspots after ‘real’ trauma, i.e., they can be described in a few words and primarily contain sensory and motion features, as well as words related to space. Our findings speak for the clinical validity of experimental trauma models (here using exposure to media footage related to the COVID-19 pandemic) to study the development of hotspots and intrusive memories in real trauma. Furthermore, our study shows that extensive exposure to e.g., COVID-19 related media footage^24^ could contribute to the development of intrusive memories in a similar way as real trauma exposure. However, future work should further explore potential differences between the content and linguistic features of hotspots and intrusive memories after analogue trauma compared to real trauma that emerged in the current study. The current study shows that a translational mental health science approach^11^ taking procedures from the clinic back to the laboratory, can not only inform theory (i.e., exploring the clinical validity of an experimental psychology model), but also reveal novel insight for future treatment development (e.g., the importance of imaginary hotspots/intrusive memories). Future work on trauma hotspots and intrusive memories, should not only include perspectives from experimental and clinical psychology, but also involve other disciplines, such as arts^25,26^ and mathematics^27^.

## Supplementary Information

Supplementary Information.
