# Supplementary material for: Qualitative analysis of hotspots and intrusive memories after viewing an aversive film highlights their sensory and spatial features
Source: Sci Rep. 2022 Apr 29;12:7049. doi: 10.1038/s41598-022-10579-0 (PMC9052176; doi:10.1038/s41598-022-10579-0)
Supplement: Supplementary file 1 — Supplementary Information. [file 41598_2022_10579_MOESM1_ESM.docx]

## Supplementary Information

Qualitative Analysis of Hotspots and Intrusive Memories After Viewing an Aversive Film Highlights Their Sensory and Spatial Features

Laura Singh, Brianna Garate, Johanna M. Hoppe, Emily A. Holmes

Scientific Reports

Doi: 10.1038/s41598-022-10579-0

Accepted 08 April 2022

De-identified summary data and codebook will be available upon publication in the Open Science Framework: https://osf.io/4zqkm/

**Supplementary Materials and Methods**

**Data analysis**

We also calculated the *number of different intrusive clips* by matching intrusive memory descriptions to the content of specific clips within the trauma film (see 2.5.5 of main manuscript). If a participant reported several intrusive memories of the same clip it was counted as one intrusive clip. To explore the overlap between clips described in hotspots and intrusive memory data, we computed the following variable for each participant included in this analysis (n = 37): the number of different intrusive clips that were also previously described as hotspots. Then, we calculated the following proportion: the total number of different intrusive clips that were previously described as hotspots across participants divided by the total number of intrusive clips reported across participants.

## Supplementary Table S1.

## Coding framework used for qualitative coding of hotspots and intrusive memories. The current framework builds on the coding frames used in Holmes et al. (2005) and Hoppe et al. (2022), but includes additional categories (e.g. imagined content) and adaptations to fit hotspots and intrusive memories of analogue trauma (i.e. ‘witnessing’ potentially traumatic events shown in a film).

| **Overarching category and code** | **Description** |
| --- | --- |
| SENSORY – VISUAL  (1= visual features present, 0=absent) | Refers to visual perceptual information and impressions. Reads like visual perceptual part of the world.  References to seeing, sight, perceiving something visually |
| SENSORY – TACTILE  (1= tactile features present, 0=absent) | Refers to tactile perceptual information, impressions and sensation. References to touching, feeling objects against body (own or others) e.g. saw cutting off limbs, COVID-19 test in nose |
| SENSORY – AUDITORY  (1= auditory features present, 0=absent) | Refers to auditory perceptual information, impressions. References to hearing or sounds. |
| NO SENSORY FEATURES  Summary variable (if none of the sensory variables are coded as 1, this category is coded as 1)  (1= no sensory features, 0= include sensory features) | No sensory features. For example, only describing thoughts or mainly describing something verbally as having happened, trying to do something (may instead be coded as cognition, cohesive narrative and/or agency category) |
| MOTION  (1= motion present, 0=absent) | Refers to motion or action, indicated by the presence of verbs associated with motion (e.g., lifting, screaming). Verbs used to describe a static scene, e.g., laying, sitting are not coded as motion (0). If it is unclear if a verb refers to motion or not, it is coded as 0. |
| BODY/BIOLOGICAL PROCESSES  (1= body/biology features present, 0=absent) | Refers to biological features, like death, blood or injury, pain or disease.  e.g. trouble breathing, death |
| CONTENT CONVEYS THREAT (1= graphic or threat-related content, 0= absent, content which out of context could describe neutral objects or events) | Refers to degree to which hotspot content is not inherently threat-related, i.e. out of context does not contain threatening elements or charged valence of emotion |
| IMAGINED CONTENT- SELF RELEVANT  (1= imagined content, self-relevant present, 0=absent) | Refers to imagined scene where participant describes events from film clip in relation to the self being present/affected e.g. “I was...” or “my mum was...” |
| IMAGINED CONTENT- ANY  (1= any imagined content present, 0= absent, content relates to film scenes) | References to any added, changed or distorted content from the film clip material where content has been imagined. |
| CRYING – SCREAMING  (1= present, 0=absent) | Presence of crying, screaming or yelling |
| EMOTION ANY  (1= present, 0=absent) | Presence of any emotion words in descriptions e.g. "fearful", “crying”. Specific emotions coded when applicable |
| EMOTION – WITNESSED  (1= present, 0=absent) | Presence of emotion words describing emotions displayed by persons in film clip. Specific emotions coded when applicable |
| EMOTION – EXPERIENCED  (1= present, 0=absent) | Presence of emotion words describing emotions experienced by the participant. Specific emotions coded when applicable |
| COGNITION ANY  (1= present, 0=absent) | Presence of cognition expressed by the participant, specified as having a thought, theme coded when applicable |

**Supplementary Results**

***Intrusive clip frequency and overlap* *of clips described in hotspots and intrusive memories***

Participants reported a mean of 2.4 different intrusive clips (*Mdn* = 2.0, *SD* = 1.9, range from 0-6) during the week. 66.3% of different discrete intrusive clips (proportion between the number of different clips that intruded and were previously described as hotspots, and the total number of different clips that intruded) referred to clips of the film that had previously been described as hotspots.

**Supplementary Table S2***.*

Overview of parts of hotspot and intrusive memory descriptions that were removed because they merely described triggers or commented a film clip (n = 8).

| Complete description with *trigger/comment*  (in italics) | Description included in analyses |
| --- | --- |
| **Hotspots** | |
| The boy who lost his childhood friend, *that was difficult to watch.* | The boy who lost his childhood friend |
| The doctor who screams on the phone at somebody. *That was probably the most difficult.* | The doctor who screams on the phone at somebody. |
| **Intrusive memories** | |
| When the woman shouts from her balcony that her mother is dying. *(Did not describe this image as one of the worst)* | When the woman shouts from her balcony that her mother is dying. |
| The face of a young chinese infront of me *when I was going to sleep and closed my eyes* | The face of a young chinese infront of me. |
| The face of the same chinese infront of me *when I was daydreaming* | The face of the same chinese infront of me. |
| *Thought about ghosts and experienced* a quick image of a chinese womans face, *same as before.* | A quick image of a chinese womans face. |
| *I thought about the film in general terms and had an intrusive memory of* the woman who screamed from her balcony about her mother. | The woman who screamed from her balcony about her mother. |
| *Diffuse memory of the film, I presume that it was images of the clip that I felt the strongest for, that is,* the last clip when people were removed from their home against their will. | The last clip when people were removed from their home against their will. |

**Supplementary Table S3***.*

Linguistic analyses’ examples of words within hotspots categorised into LIWC

word categories of interest

| *LIWC^1^ word category* | All coded words |
| --- | --- |
| - **Relativity** |  |
| - Motion - Space - Time | car, leave, removed, pushed  on, out, around, toward, floor, street  when, anymore, young, waiting, after |
| **Perception**   - See - Hear - Feel | see, light, image, looked  screaming, shouts, yelled, phone, saying  pain, skin, tightly |
| - **Biological processes** |  |
| - Body - Health - Sexual - Ingestion | head, bodies, throat, skin, face, breathing, lungs  hospital, sick, nurse, doctor, pain, infected, illness, ICU, live  naked  Pepsi, lunch, dining, vodka, drinks, liquor |
| - **Time orientation** |  |
| - Past focus - Present focus - Future focus | was, tried, sat, yelled, died, had  is, are, lay, can, does, get, talks, have, comes  then, will, going, prepared |
| **Affective processes** |  |
| - Positive - Negative - Anxiety - Anger - Sadness | partying, parties, helping, liked, healthy, care, laugh  ignored, screaming, crying, sick, pressure, panic  terror, pressure, panic, desperate, worries  violent, yelled, threatens, attack  crying, grief, abandoned, lone, despair, hopeless, lost |
| **Cognitive processes** |  |
| - Insight - Causation - Discrepancy - Tentative - Certainty - Differentiation | thought, memory, suspected, felt, seems  force, because, how, results  needed, want, wished, could  or, if, some, anywhere, most, likely, seems, potential  particularly, all, everybody, completely, clear, entire  against, not, or, If, but, without, instead, despite |

*Note*. Linguistic analysis was conducted with the ^1^ Linguistic inquiry and word count

(LIWC 2015 for Windows; Pennebaker et al., 2015; Tausczik & Pennebaker, 2010).

**Supplementary Table S4***.*

Linguistic analyses’ examples of words within intrusive memories categorised into LIWC

word categories of interest

| *LIWC^1^ word category* | All coded words |
| --- | --- |
| **Relativity** |  |
| - Motion - Space - Time | transported, carried, going, removed, turn  out, in, street, at, floor, into, away, where, room, site  again, young, year, old, when, back, waiting |
| **Perception**   - See - Hear - Feel | image, saw, black  silences, screaming, shouts, phone, spoke  feeling |
| - **Biological processes** |  |
| - Body - Health - Sexual - Ingestion | bodies, breathing, head, face  hospital, sick, doctor, nurse  -  Pepsi, dining |
| - **Time orientations** |  |
| - Past focus - Present focus - Future focus | ignored, died, had, passed, cried, played  is, has, tells, die, can, turn  going, will |
| **Affective processes** |  |
| - Positive - Negative - Anxiety - Anger - Sadness | party, respect  ignored, screaming, cry, alone, panic, difficulties, sick, violent, struggles  panic, struggles  violent  cry |
| **Cognitive processes** |  |
| - Insight - Causation - Discrepancy - Tentative - Certainty - Differentiation | feeling, memory, explained, seem, imagine  because, how, forced  -  kind of, seem, any, perhaps, some, most likely  -  but, without, not, against, or |

*Note*. Linguistic analysis was conducted with the ^1^ Linguistic inquiry and word count

(LIWC 2015 for Windows; Pennebaker et al., 2015; Tausczik & Pennebaker, 2010).

**References**

Holmes, E. A., Grey, N., & Young, K. A. D. (2005). Intrusive images and “hotspots” of trauma memories in Posttraumatic Stress Disorder: an exploratory investigation of emotions and cognitive themes. *Journal of Behavior Therapy and Experimental Psychiatry*, *36*(1), 3–17. https://doi.org/10.1016/j.jbtep.2004.11.002

Hoppe, J. M., Walldén, Y. S. E., Kanstrup, M., Singh, L., Agren, T., Holmes, E. A., & Moulds, M. L. (2022). Hotspots in the Immediate Aftermath of Trauma - Mental Imagery of Worst Moments Highlighting Time, Space and Motion. *Consciousness & Cognition*, *99*, 103286, https://doi.org/10.1016/j.concog.2022.103286.

Pennebaker, J. W., Boyd, R. L., Jordan, K., & Blackburn, K. (2015). *​ The development and psychometric properties of LIWC2015​*. https://repositories.lib.utexas.edu/handle/2152/31333

Tausczik, Y. R., & Pennebaker, J. W. (2010). The psychological meaning of words: LIWC and computerised text analysis methods. *Journal of Language and Social Psychology*, *29*(1), 24–54. https://doi.org/10.1177/0261927X09351676
